# Trends in Gender Authorship and Collaborations: A 30-Year Comparative Bibliometric Analysis of Manuscripts from The Journal of Bone and Joint Surgery and The Bone and Joint Journal

**DOI:** 10.1155/2020/5019607

**Published:** 2020-12-18

**Authors:** Maria E. Squire, Katherine Schultz, Donnell McDonald, Cory Meixner, Dayton Snyder, Alyssa M. Cooke, Jacob C. Davis, Sarina Masso Maldonado, Carlos R. Martinez Licha, Elizabeth C. Whipple, Melissa A. Kacena, Randall T. Loder

**Affiliations:** ^1^University of Scranton, Department of Biology, Scranton, PA, USA; ^2^Indiana University School of Medicine, Department of Orthopaedic Surgery, Indianapolis, IN, USA; ^3^Ruth Lilly Medical Library, Indiana University School of Medicine, Indianapolis, IN, USA

## Abstract

Publishing original peer-reviewed research is essential for advancement through all career stages. Fewer women than men hold senior-level positions in academic medicine and, therefore, examining publication trends relative to gender is important. The goal of this study was to examine and compare publication trends in *The Journal of Bone and Joint Surgery* (*JBJS*) and *The Bone and Joint Journal* (*BJJ*) with a particular emphasis on trends regarding author gender. Data was collected and analyzed for manuscripts published in *JBJS* and *BJJ* over the past 30 years. For manuscripts published in 1986, 1996, 2006, and 2016, we recorded the numbers of authors, manuscript pages, references, collaborating institutions, the position in the byline of the corresponding author, the country of the corresponding author, and the names of the first and corresponding author. We also calculated the normalized number of citations and corresponding author position. The number of authors, institutions, and countries collaborating on manuscripts published in both *JBJS* and *BJJ* increased over time. *JBJS* published more manuscripts from North America and *BJJ* published more manuscripts from Europe. In both journals, the percentage of women as first and/or corresponding author increased over time. Trends over the past 30 years have shown increased collaborations with greater citations in manuscripts published in *JBJS* and *BJJ*. In the same time period, both journals demonstrated a rise in the percentage of manuscripts with women first and/or corresponding authors, suggesting a decrease in the gender gap.

## 1. Introduction

Original research in peer-reviewed journals allows the review and dissemination of new information. The increasing complexity of research has resulted in increased collaboration among researchers (same institution or geographic region, or different countries) to obtain appropriate expertise [1–3]. Collaboration is easier with the advances in technology [3]. Studies have suggested that international collaborations expand the dissemination of information, increasing readership and citations [1, 4]. Increasing collaboration may also be due to the “publish or perish” paradigm [5–9], as publication production is important at all career stages [9–11].

Medicine has traditionally been a field dominated by men, although women have made significant gains; in 2018-2019, women represented 49.5% of US medical school matriculates [12]. Due to a few senior-level women in academic medicine, it is important to examine publication trends relative to gender. In the USA, there has been no meaningful change in orthopedic surgery residents from 10.9% in 2006 to 14.4% in 2015 [13]. This minimal change likely reflects the minimal change in the percentage of women graduates in US medical schools, 41.5% in 2006 and 47.4% in 2016 [14]. In the UK, women comprised 25% of orthopedic trainees and only 5% of all orthopedic surgery consultants in 2014 [15]. In spite of attempts to close the gender gap [16], women still lag behind men. The goals of this study were twofold: (1) to examine publication trends in *The Journal of Bone and Joint Surgery* (*JBJS*) and *The Bone and Joint Journal* (*BJJ*) in bibliometric variables over time and (2) specifically to study author gender trends. These two journals were chosen as they are well-known orthopedic surgery journals covering material from all orthopedic subspecialties and highly regarded in both the English and non-English speaking world. As *JBJS* is a US-based journal and *BJJ* a UK-based journal, it also allows for studying author gender trends between the USA and UK. Such data will be helpful for trainees, junior faculty, and senior faculty in mentorship positions.

## 2. Materials and Methods

Manuscripts published in *JBJS* and *BJJ* over the past 30 years were analyzed using a well-established method [17]. Data was collected for 1986, 1996, 2006, and 2016. Excluded from the analysis were editorials, memorandums, letters, commentaries, and case reports. The number of authors, manuscript pages, references, and collaborating institutions, corresponding author position (CAP) in the byline, country of the corresponding author, and names of the first and corresponding author were collected. A Scopus search performed in December of 2017 determined the number of times each manuscript had been cited and was normalized by dividing by the age of the manuscript in years. The CAP was standardized by dividing by the total number of authors on the manuscript. The corresponding author's geographical location was categorized into regions: Asia, Australia/New Zealand, Europe, Latin America, and North America.

The first and corresponding author gender was determined using the “Baby Name Guesser” software of Geoff Peters' (http://www.gpeters.com/names/baby-names.php). The “Baby Name Guesser” website produces a gender and gender ratio for first names. This method was previously used in several bibliometric studies from our group [17–26] as well as others in both medicine [27–31] and other disciplines [32, 33]. For a ratio ≥3.0, the gender given was assumed correct. If below 3.0, the gender was manually verified using a Google search. If no gender could be ascertained, then the manuscript was excluded from gender analyses.

### 2.1. Statistical Analyses

Discrete data are reported as frequencies and percentages and continuous data as the mean ± 1 standard deviation. Differences in categorical data were analyzed using Fisher's exact test (2 × 2 tables) or Pearson's *χ*2 test (>2 × 2 tables). Nonparametric tests were used to analyze differences in continuous data due to nonnormal distributions (Mann–Whitney *U* for 2 groups; Kruskal–Wallis test for 3 or more groups). Observed trends over the 10-year intervals were analyzed using the Cochran linear trend test. Statistical analyses were performed with Systat 10 software™ (Systat Software, Chicago, IL, 2000) with a significance level of 0.05.

## 3. Results

### 3.1. Cross-Journal Analysis

There were 989 *JBJS* and 893 *BJJ* manuscripts; *JBJS* manuscripts had more authors (4.6 ± 2.3 vs. 4.3 ± 2.1, *p* = 0.005), references (30.5 ± 22.1 vs. 25.7 ± 18.0, *p* < 10^−6^), printed pages (8.0 ± 2.8 vs. 5.5 ± 2.0, *p* < 10^−6^), and normalized citations (4.56 ± 5.29 vs. 3.07 ± 2.46, *p* < 10^−6^) than *BJJ* manuscripts. There were no differences between journals for standardized CAP (0.43 ± 0.32 vs. 0.43 ± 0.30, *p* = 0.10), the number of collaborating institutions (2.2 ± 1.9 vs. 2.0 ± 1.4, *p* = 0.65), countries (1.2 ± 0.6 vs. 1.2 ± 0.5, *p* = 0.20), or single author manuscripts (49 vs. 55, *p* = 0.27).

### 3.2. Analyses by Region

The regions were known for 987 *JBJS* and 882 *BJJ* manuscripts. For *JBJS*, the manuscripts came from North America (*n* = 758), Europe (*n* = 158), Asia (*n* = 61), and Australia/New Zealand (*n* = 10). For *BJJ*, the manuscripts came from Europe (*n* = 587), North America (*n* = 128), Asia (*n* = 118), and Australia/New Zealand (*n* = 49).

For *JBJS* (Table 1), manuscripts with the greatest number of authors came from Australia/New Zealand (5.8 ± 2.6) and the least from North America (4.4 ± 2.4). For *BJJ*, manuscripts with the greatest number of authors came from Asia (4.8 ± 1.9) and the least from Australia/New Zealand (4.2 ± 1.9). Manuscripts with the greatest standardized CAP, for both *JBJS* and *BJJ*, came from Australia/New Zealand (0.56 ± 0.43 and 0.54 ± 0.35) followed by North America (0.45 ± 0.33 and 0.50 ± 0.32). For *JBJS*, the lowest standardized CAP was for manuscripts from Europe (0.37 ± 0.30) whereas for *BJJ* it was the lowest for those from Asia (0.41 ± 0.30). There was a difference for *JBJS* in the number of collaborating countries between North America (1.1 ± 0.5), Europe (1.4 ± 1.0), Asia (1.1 ± 0.2), and Australia/New Zealand (1.7 ± 0.7), but there was no difference for *BJJ*. There were no statistically significant differences in the number of institutions or printed pages. There were no differences in the number of normalized citations for *JBJS*; there was a difference for *BJJ* with the highest from North America (3.48 ± 3.67) and Asia the lowest (1.90 ± 1.92). There were no regional differences in the number of references for *JBJS*; in *BJJ*, the most came from North America (30.5 ± 17.1), and the least from Europe (24.8 ± 18.6).

### 3.3. Analyses Over Time by Journal

**Figure 1:**
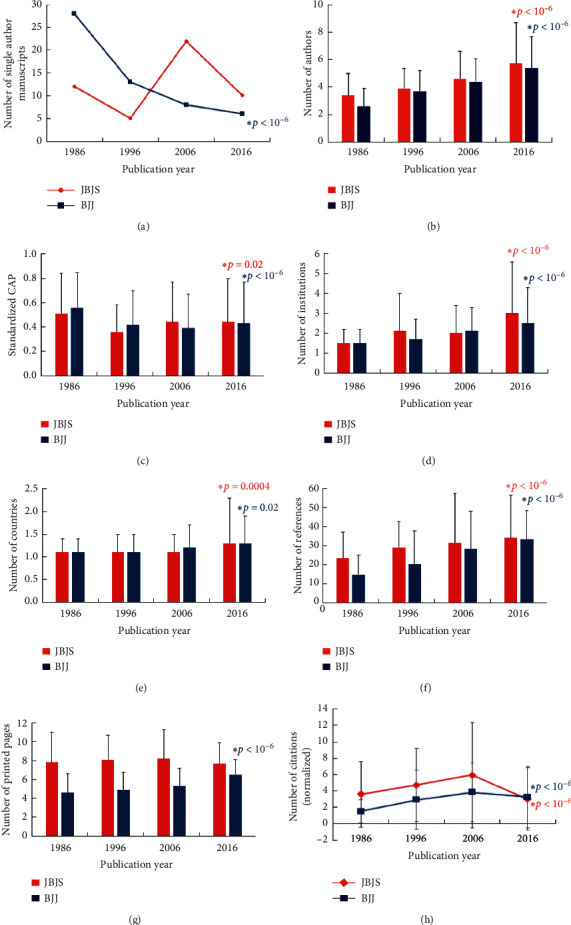
Analyses by geographic region over time for *JBJS* and *BJ*J for (a) the number of single author manuscripts, (b) number of authors over time in *JBJS* and *BJJ*, (c) standardized corresponding author position (CAP), (d) number of collaborating institutions, (e) number of countries, (f) number of references, (g) number of printed pages, and (h) number of normalized citations. The asterisks represent those having statistically significant differences over the four decades as assessed by the Cochran linear trend test.

The number of manuscripts published increased from 1986 to 2006 before declining in 2016. For *JBJS,* there were 154 in 1986, 159 in 1996, 400 in 2006, and 276 in 2016. For *BJJ,* there were 159 in 1986, 178 in 1996, 298 in 2006, and 258 in 2016. The number of single-authored manuscripts in *JBJS* did not show a significant change (*p* = 0.17) but declined in *BJJ* (*p* < 10^−6^) (Figure 1(a)). The number of authors increased significantly in both *JBJS* (*p* < 10^−6^) and *BJJ* (*p* < 10^−6^) (Figure 1(b)). The standardized CAP changed over time for both *JBJS* (*p* = 0.02) and *BJJ* (*p* < 10^−6^) (Figure 1(c)). The number of collaborating institutions increased significantly in *JBJS* (*p* < 10^−6^) and *BJJ* (*p* < 10^−6^) (Figure 1(d)) as did the number of countries for *JBJS* (*p* = 0.0004) and *BJJ* (*p* = 0.02) (Figure 1(e)). For both journals, the number of references increased from 1986 to 2016 (*p* < 10^−6^) (Figure 1(f)). While the number of printed pages did not change in *JBJS* (*p* = 0.30), there was a 41% increase in the number of printed pages from 1986 to 2016 in *BJJ* (*p* < 10^−6^) (Figure 1(g)). The number of normalized citations increased in *JBJS* from 3.6 ± 4.0 in 1986 to 5.9 ± 6.4 in 2006 and then decreased to 3.0 ± 3.8 in 2016 (*p* < 10^−6^) (Figure 1(h)); for *BJJ*, it increased from 1.5 ± 1.4 in 1986 to 3.9 ± 3.6 in 2006 and then decreased to 3.3 ± 3.8 in 2016 (*p* < 10^−6^) (Figure 1(h)).

### 3.4. Analyses by Gender

First author gender was available for 97.0% of *JBJS* and 90.6% of *BJJ* manuscripts. For *JBJS*, 11.5% of first authors were women and 10.6% for *BJJ* (*p* = 0.60). Corresponding author gender was available for 84.7% of *JBJS* and 93.2% of *BJJ* manuscripts. In *JBJS*, 10.9% of the corresponding authors were women and 9.1% for *BJJ* (*p* = 0.25).

There were no differences by first author gender (Table 2) in *JBJS* for the numbers of authors, institutions, collaborating countries, normalized citations, references, printed pages, or standardized CAP. The findings were the same in *BJJ* except for the number of printed pages; manuscripts with women first authors were longer (6.4 ± 2.2) than those with men first authors (5.4 ± 1.9).

There were no differences by corresponding author gender (Table 3) for the numbers of authors, institutions, collaborating countries, normalized citations, references, printed pages, or standardized CAP for *JBJS*. Findings were the same for *BJJ*, except for the number of references and printed pages. *BJJ* manuscripts with women corresponding authors were longer (6.3 ± 2.2) than those with men corresponding authors (5.4 ± 1.9) with more references (29.6 ± 17.6, 25.8 ± 17.8).

### 3.5. Analyses by Gender across Regions

The percentage of women first authors in *JBJS* manuscripts for all four decades varied by region with Australia/New Zealand having the most (30%), then Europe (13%), North America (11.6%), and Asia (2%) (*p* = 0.046). The percentage of manuscripts with women first authors in *BJJ* did not vary by region (Australia/New Zealand (13%), Europe (11.4%), Asia (8.4%), and North America (8.1%) (*p* = 0.60)).

### 3.6. Analyses by Gender over Time

**Figure 2:**
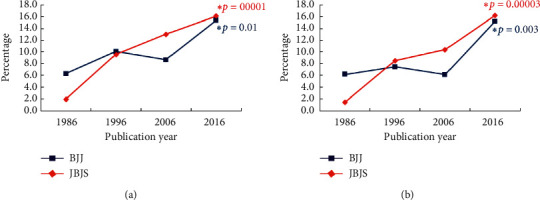
Percentage of women as first (a) or corresponding (b) authors over time in *JBJS* and *BJJ*. The asterisks represent those having statistically significant changes over the four decades as assessed by the Cochran linear trend test.

The percentage of manuscripts with women first authors increased from 2.0% to 16.1% in *JBJS* (*p* = 0.00001) and from 6.3% to 15.3% in *BJJ* (*p* = 0.01) (Figure 2(a)). The percentage of manuscripts with women corresponding authors increased from 1.4% to 16.2% in *JBJS* (*p* = 0.0003) and from 6.2% to 15.2% in *BJJ* (*p* = 0.003) (Figure 2(b).

**Figure 3:**
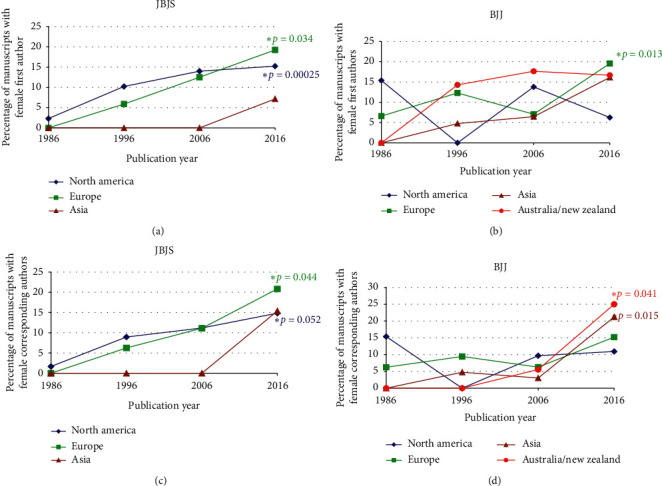
Changes in the percentage of women as the first author for (a) *JBJS* and (b) *BJJ* and women as the corresponding author for (c) *JBJS* and (d) *BJJ*. The asterisks represent those having statistically significant changes over the four decades as assessed by the Cochran linear trend test.

We also analyzed differences in author gender over time by region (Table 4). Due to the small number from Australia/New Zealand, those were excluded for *JBJS*. For *JBJS*, the percentage of manuscripts with women first authors (Figure 3(a)) rose from 2.3% to 15.3% (*p* = 0.0025) for those from North America, from 0.0% to 19.2% (*p* = 0.034) for those from Europe, and with no significant change in those from Asia. For *BJJ*, the percentage of manuscripts with women first authors (Figure 3(b)) rose from 6.6% to 19.6% (*p* = 0.013) for those from Europe, with no significant changes in those from North America, Australia/New Zealand, or Asia. For *JBJS*, the percentage of manuscripts with women corresponding authors (Figure 3(c)) rose from 1.7% to 14.8% (*p* = 0.0052) for those from North America and 0.0% to 20.8% (*p* = 0.044) for those from Europe, with no significant changes for those from Asia. For *BJJ*, the percentage of manuscripts with women corresponding authors (Figure 3(d)) rose from 0.0% to 21.2% (*p* = 0.015) for those from Asia and from 0.0% to 25.0% (*p* = 0.041) for those from Australia/New Zealand, with no significant changes for those from North America or Europe.

### 3.7. Analyses by Gender across Subspecialties

**Figure 4:**
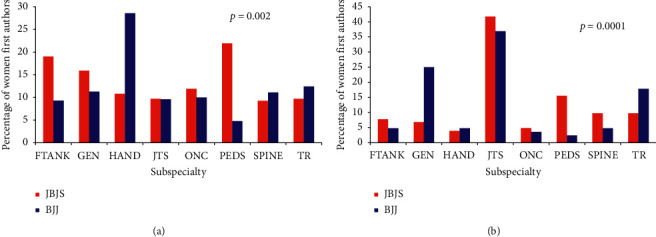
Percentage of women first authors across orthopedic subspecialties in *JBJS* and *BJJ*. Reporting percentages of women first authors relative to (a) the total number of manuscripts in a subspecialty and (b) the total number of manuscripts across all subspecialties. FTANK = foot and ankle, GEN = general, JTS = joint arthroplasty, ONC = oncology, PEDS = pediatrics, and TR = trauma.

Manuscripts were categorized by subspecialty into foot and ankle, general, hand, joint arthroplasty, oncology, pediatrics, spine, sports medicine, and trauma (Figure 4). The sports medicine subspecialty was excluded from the analysis due to low numbers (15 from *JBJS* and one from *BJJ*). Reporting percentages of women first authors relative to the total manuscripts within each subspecialty (Figure 4(a)), the largest percentage of women first authors in *JBJS* was in pediatrics (22%) and the lowest in the spine (9%). In *BJJ*, the largest percentage of women first authors was in hand (29%) and the lowest in pediatrics (5%). These differences between journals were significant (*p* = 0.002). Reporting percentages of women first authors in a subspecialty relative to the total manuscripts across all subspecialties (Figure 4(b)), the largest percentage of women first authors in both journals was in joint arthroplasty (42% for *JBJS* and 37% for *BJJ*). The second highest was in pediatrics for *JBJS* (16%) and general for *BJJ* (25%); the lowest percentage of women first authors was in hand for *JBJS* (4%) and in pediatrics for *BJJ* (2%) (*p* = 0.0014).

### 3.8. Analyses by Author Gender Combination over Time

**Figure 5:**
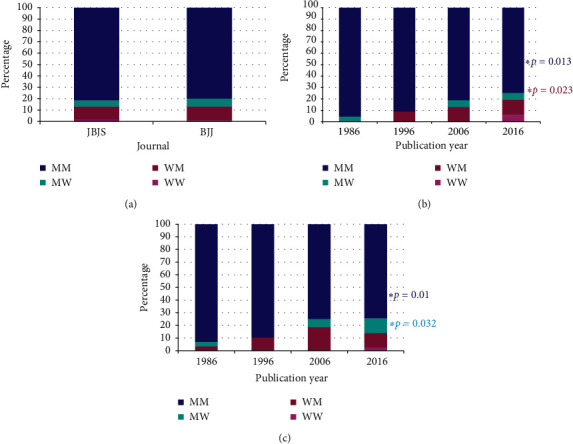
Gender combinations of first and corresponding authors for *JBJS* and *BJJ*. MM = men as first and corresponding authors, MW = men as first and women as corresponding authors, WM = women as first and men as corresponding authors, WW = women as first and corresponding authors. (a) Between journals, (b) for JBJS, and (c) for BJJ. The asterisks represent those having statistically significant changes over the four decades as assessed by the Cochran linear trend test.

Manuscripts were categorized into one of four gender combinations between the first author and corresponding author: WW (women as first and corresponding authors), WM (women as first and men as corresponding authors), MW (men as first and women as corresponding authors), and MM (men as first and corresponding authors). Single author manuscripts and those where the first author was also the corresponding author were excluded from these analyses. There were no significant differences in any of the four gender combinations between journals (Figure 5(a)) (*p* = 0.80). There were significant differences over time for two gender combinations for *JBJS* (Figure 5(b)): WW (*p* = 0.023) and MM (*p* = 0.013), but not WM (*p* = 0.10) or MW (*p* = 0.51). There were significant differences over time for two gender combinations for *BJJ* (Figure 5(c)): MW (*p* = 0.032) and MM (*p* = 0.01), but not WW (*p* = 0.15) or WM (*p* = 0.30).

## 4. Discussion

The number of authors, institutions, and countries collaborating on manuscripts published in both journals increased from 1986 to 2016. The increase in author number may be due to the need for individuals with different skill sets/expertise due to increasing research complexity [1–3]. Similar increases in author number have been reported across several musculoskeletal journals [17–26]. This increase may also be tied to the pressure for publication at all career levels [7, 34, 35]. Advances in technology and communication have made long-distance and intercountry collaborations more feasible [4]. The increase in collaboration seen over time in *JBJS* and *BJJ* has likely also contributed to the increase in the number of citations [1, 36]. The number of authors and pages in both *JBJS* and *BJJ* may decrease in the future, as both journals have implemented word and author number limits; 3000 words (excluding references) and no more than 6 authors for JBJS [37] and 4000 words (including references) and no more than 8 authors for BJJ [38]. However, throughout the time span of this study, there was a gradual increase in the number of references and authors for both *JBJS* and *BJJ* and manuscript length for *BJJ*.

The number of manuscripts published in both *JBJS* and *BJJ* increased from 1986 to 2006 before decreasing in 2016. The largest number of manuscripts for *JBJS* came from North America and for *BJJ* from Europe. This is expected as authors from North America would likely favor *JBJS* and European investigators *BJJ*. It is not clear why the number of manuscripts in both journals increased up to 2006 and then dropped in 2016. Possible explanations are the increasing number of subspecialty journals as well as the introduction of several other journals under the umbrella of *JBJS* and *BJJ*.

One goal of this study was to examine the author's gender over time. In both journals, there was an increase in the percentage of women first and corresponding authors, but it was lower than the percentages of women US medical school graduates (50%) [39] and women accepted to medical school in the UK (58%) [40]. In 2013, in the US, women comprised 13.7% of orthopedic residents [41]. In 2016, women comprised only 6.5% of the American Academy of Orthopaedic Surgeons membership [42]. In 2014, in the UK, women comprised 25% of orthopedic trainees and 5% of orthopedic surgery consultants [15]. This may partly explain our findings regarding author gender.

We next began to explore possible mentorship trends and gender in authorship. To accomplish this, we used first and corresponding author gender composition ratios as a surrogate for mentorship, making the assumption that when first and corresponding authors were different individuals, the first author is the mentee of the corresponding author. With this in mind, WW gender combinations for first and corresponding authors increased in JBJS with time, and 2016 was also the first time that WW gender combinations were present in both *JBJS* and *BJJ*. This suggests an increase in the overall percentage of women, early in their careers, serving as the first author and being mentored by more senior women. This likely reflects the fact that over the last ten years women have now become President of the British Orthopaedic Association (2008), Royal College of Surgeons (2014), and American Academy of Orthopaedic Surgeons (2019). As women take on more leadership positions and mentor more junior women, the percentages of women first and corresponding authors will likely continue to increase. Another interesting observation was that while the overall gender combinations were the same between *JBJS* and *BJJ*, when looking at trends over time, there were significant differences. In both journals, MM combinations were reduced with time, but which categories were responsible for the reduction varied. For *JBJS,* there was a significant increase in the percentage of WW authors, whereas, for *BJJ,* there was a significant increase in the percentage of MW authors. While not conclusively tested here, these findings may indicate that for *JBJS*, and the North American region by proxy, the increase in WW authors is likely a reflection of the increase in women orthopedic surgeons in the field, both at the more senior levels and at the trainee/junior levels. Perhaps, more interesting is the increase in MW combinations observed in *BJJ*. There are a number of ways this data can be interpreted, but it may reflect a larger change in approach/attitude toward mentee/mentorship. This may suggest that for *BJJ* (and Europe by proxy), more junior men are seeking women as mentors or that women are seeking to serve as mentors to more men. This may be a positive sign that change is on the horizon when considering the gender gap in authorship.

Although there are fewer women orthopedic surgeons compared to men, studies have shown that women choose particular orthopedic subspecialties more than others [43, 44]. In the current study, we found that the greatest percentage of manuscripts published by women was in joint arthroplasty. This is surprising as only 8.7% of women orthopedic surgeons in the USA select joint arthroplasty as their subspecialty [44]. The fact that joint arthroplasty manuscripts were the most common for women first authors in both journals may have several explanations. One explanation could be that joint arthroplasty may simply represent the subspecialty area with the greatest number of submitted/accepted manuscripts. Indeed, joint arthroplasty manuscripts accounted for 49% and 39% of all manuscripts published in the *JBJS* and *BJJ* during the years examined in the current study. Outside of joint arthroplasty, the greatest percentage of manuscripts with women as first authors was in pediatric orthopedics for JBJS and general for BJJ when considering women first authors across all subspecialties and in pediatric orthopedics and hand for JBJS and BJJ, respectively, when considering the total number of manuscripts in a particular subspecialty. This corroborates a 2016 survey of Ruth Jackson Orthopaedic Society women members which reported larger percentages of women surgeons listing hand (22%) and pediatrics (16%) as their subspecialty. In our study, the lowest percentage of manuscripts with women first authors for *JBJS* was the hand (3.9%) and for *BJJ* was pediatrics (2.4%) when considering women first authors across all subspecialties and the lowest were in the spine and pediatric orthopedics in *JBJS* and *BJJ*, respectively, when considering the total number of manuscripts in a particular subspecialty. While the finding in *JBJS* correlates with the 2016 survey reporting that women comprised the highest percentage of fellowship applicants for pediatrics (25%), the *BJJ* finding that pediatric subspecialty manuscripts had few women first authors was surprising; this may reflect continental differences in subspecialty selection.

## 5. Conclusion

Manuscripts in *JBJS* and *BJJ* over the past 30 years have shown increases in the number of collaborating institutions, collaborating countries, references, and citations. The gender gap has decreased with the percentage of manuscripts with women first and/or corresponding authors increasing over the past 30 years.

## Figures and Tables

**Table 1:** Bibliometric analyses by journal and geographical region.

	*JBJS*					*BJJ*				
	North America (*n* = 758)	Europe (*n* = 158)	Asia (*n* = 61)	Australia/New Zealand (*n* = 10)	*p* value	North America (*n* = 128)	Europe (*n* = 587)	Asia (*n* = 118)	Australia/New Zealand (*n* = 49)	*p* value
Author no.	4.4 ± 2.4	4.9 ± 2.4	5.4 ± 1.7	5.8 ± 2.6	0.000009	4.3 ± 2.1	4.2 ± 2.1	4.8 ± 1.9	4.2 ± 1.9	0.004
No. of institutions	2.2 ± 1.9	2.3 ± 2.0	2.1 ± 1.3	3.5 ± 2.8	0.28	2.1 ± 1.4	2.9 ± 1.4	1.9 ± 1.2	2.2 ± 1.5	0.44
No. of countries	1.1 ± 0.5	1.4 ± 1.0	1.1 ± 0.2	1.7 ± 0.7	<10^−6^	1.2 ± 0.5	1.2 ± 0.5	1.1 ± 0.3	1.2 ± 0.5	0.072
No. of citations (normalized)	4.44 ± 5.04	5.67 ± 6.67	3.18 ± 3.51	4.67 ± 5.53	0.083	3.48 ± 3.67	3.20 ± 3.63	1.90 ± 1.92	3.49 ± 3.47	0.0003
No. of references	31.0 ± 23.6	30.5 ± 16.8	24.7 ± 11.3	34.6 ± 24.2	0.24	30.5 ± 17.1	24.8 ± 18.6	25.3 ± 16.3	26.5 ± 16.2	0.0001
No. of pages	7.9 ± 3.0	7.9 ± 2.4	7.8 ± 2.2	9.4 ± 0.8	0.15	5.5 ± 1.7	5.4 ± 2.0	5.7 ± 1.9	5.7 ± 2.0	0.28
Corresponding author position (standardized)	0.45 ± 0.33	0.37 ± 0.30	0.40 ± 0.32	0.56 ± 0.43	0.046	0.50 ± 0.32	0.42 ± 0.29	0.41 ± 0.30	0.54 ± 0.35	0.009

Values are mean ± 1 standard deviation.

**Table 2:** Bibliometric analyses by journal and first author gender.

	*JBJS*			*BJJ*		
	Woman	Man	*p* value	Woman	Man	*p* value
Author number	4.7 ± 2.5	4.6 ± 2.3	0.81	4.7 ± 2.1	4.3 ± 2.1	0.11
Number of institutions	2.3 ± 1.6	2.2 ± 1.9	0.12	2.3 ± 1.4	2.0 ± 1.4	0.21
Number of countries	1.2 ± 0.5	1.2 ± 0.6	0.38	1.2 ± 0.4	1.2 ± 0.5	0.47
Number of citations (normalized)	5.07 ± 4.86	4.53 ± 5.34	0.073	4.01 ± 4.86	3.09 ± 3.28	0.18
Number of references	30.4 ± 18.7	30.4 ± 22.6	0.61	28.6 ± 16.6	26.2 ± 18.2	0.08
Number of pages	7.8 ± 2.5	8.0 ± 2.9	0.57	6.4 ± 2.2	5.4 ± 1.9	0.00012
Corresponding author position (standardized)	0.45 ± 0.34	0.43 ± 0.32	0.69	0.45 ± 0.31	0.41 ± 0.29	0.59

Values are mean ± 1 standard deviation.

**Table 3:** Bibliometric analyses by journal and corresponding author gender.

	*JBJS*			*BJJ*		
	Woman	Man	*p* value	Woman	Man	*p* value
Author number	4.8 ± 2.2	4.6 ± 2.3	0.53	4.7 ± 2.2	4.3 ± 2.1	0.14
Number of institutions	2.3 ± 1.5	2.3 ± 2.0	0.26	2.2 ± 1.5	2.0 ± 1.4	0.60
Number of countries	1.1 ± 0.4	1.2 ± 0.7	0.56	1.1 ± 0.3	1.2 ± 0.5	0.097
Number of citations (normalized)	4.87 ± 5.04	4.74 ± 5.51	0.71	3.52 ± 3.64	3.16 ± 3.53	0.56
Number of references	29.9 ± 15.9	31.1 ± 22.8	0.89	29.6 ± 17.6	25.8 ± 17.8	0.02
Number of pages	7.8 ± 2.3	8.0 ± 2.9	0.60	6.3 ± 2.2	5.4 ± 1.9	0.0007
Corresponding author position (standardized)	0.39 ± 0.29	0.44 ± 0.33	0.20	0.39 ± 0.29	0.43 ± 0.31	0.18

Values are mean ± 1 standard deviation.

**Table 4:** Bibliometric analyses by author gender, journal, and geographic region over time.

	First author									Corresponding author								
	*n*				% *n*					*n*				% *n*				
	1986	1996	2006	2016	1986	1996	2006	2016	*p* value^∧^	1986	1996	2006	2016	1986	1996	2006	2016	*p* value^∧^
*JBJS* and *BJJ*																		
All woman	11	32	74	78	3.9	9.8	11.2	15.8	0.000001	9	26	58	74	4.5	8.0	8.7	15.8	0.000003
Man	268	293	586	415	96.1	90.2	88.8	84.2		189	301	608	394	95.5	92.0	91.3	84.2	
North America woman	5	14	46	31	3.5	9.0	14.0	12.9	0.002	3	12	36	30	4.1	7.8	11.0	13.7	0.01
Man	138	141	283	210	96.5	91.0	86.0	87.1		70	141	290	189	95.9	92.2	89.0	86.3	
Europe woman	6	16	22	37	5.8	11.5	8.6	19.5	0.0014	6	13	20	31	5.9	9.1	7.6	16.7	0.005
Man	98	123	234	153	94.2	88.5	91.4	80.5		96	130	243	155	94.1	90.9	92.4	83.3	
Asia woman	0	1	2	6	0.0	4.3	3.6	13.3	0.036	0	1	1	9	0.0	4.3	1.8	19.6	0.006
Man	20	22	54	39	100.0	95.7	96.4	86.7		13	22	56	37	100.0	95.7	98.2	80.4	
Aus/NZ woman	0	1	4	4	0.0	12.5	21.1	23.5	0.076	0	0	1	4	0.0	0.0	5.0	23.5	0.028
Man	12	7	15	13	100.0	87.5	78.9	76.5		10	8	19	13	100.0	100.0	95.0	76.5	

*JBJS*																		
All woman	3	15	52	40	2.0	9.6	13.0	16.1	0.00001	1	13	41	36	1.4	8.5	10.4	16.3	0.0003
Man	150	142	347	208	98.0	90.4	87.0	83.9		68	140	352	185	98.6	91.5	89.6	83.7	
North America woman	3	14	42	27	2.3	10.2	14.0	15.3	0.00025	1	12	33	23	1.7	9.0	11.2	14.8	0.0052
Man	127	123	258	150	97.7	89.8	86.0	84.7		59	122	262	132	98.3	91.0	88.8	85.2	
Europe wman	0	1	9	10	0.0	5.9	12.5	19.2	0.034	0	1	8	10	0.0	6.3	11.1	20.8	0.044
Man	13	16	63	42	100.0	94.1	87.5	80.8		6	15	64	38	100.0	93.8	88.9	79.2	
Asia woman	0	0	0	1	0.0	0.0	0.0	7.1	0.27	0	0	0	2	0.0	0.0	0.0	15.4	0.10
Man	8	2	25	13	100.0	100.0	100.0	92.9		2	2	24	11	100.0	100.0	100.0	84.6	

*BJJ*																		
All woman	8	17	22	38	6.3	10.1	8.4	15.5	0.01	8	13	17	38	6.2	7.5	6.2	15.4	0.003
Man	118	151	239	207	93.7	89.9	91.6	84.5		121	161	256	209	93.8	92.5	93.8	84.6	
North America woman	2	0	4	4	15.4	0.0	13.8	6.3	0.60	2	0	3	7	15.4	0.0	9.7	10.9	0.72
Man	11	18	25	60	84.6	100.0	86.2	93.8		11	19	28	57	84.6	100.0	90.3	89.1	
Europe woman	6	15	13	27	6.6	12.3	7.1	19.6	0.013	6	12	12	21	6.3	9.4	6.3	15.2	0.051
Man	85	107	171	111	93.4	87.7	92.9	80.4		90	115	179	117	93.8	90.6	93.7	84.8	
Asia woman	0	1	2	5	0.0	4.8	6.5	16.1	0.06	0	1	1	7	0.0	4.8	3.0	21.2	0.015
Man	12	20	29	26	100.0	95.2	93.5	83.9		11	20	32	26	100.0	95.2	97.0	78.8	
Aus/NZ woman	0	1	3	2	0.0	14.3	17.6	16.7	0.23	0	0	1	3	0.0	0.0	5.6	25.0	0.041
Man	10	6	14	10	100.0	85.7	82.4	83.3		9	7	17	9	100.0	100.0	94.4	75.0	

^ Cochran linear trend test.

Aus/NZ = Australia/New Zealand.

## Data Availability

The data are available from the corresponding author upon request.
